# Stent thrombosis rates the first year and beyond with new- and old-generation drug-eluting stents compared to bare metal stents

**DOI:** 10.1007/s00392-018-1252-0

**Published:** 2018-04-17

**Authors:** Christoph Varenhorst, Martin Lindholm, Giovanna Sarno, Göran Olivecrona, Ulf Jensen, Johan Nilsson, Jörg Carlsson, Stefan James, Bo Lagerqvist

**Affiliations:** 1 Uppsala Clinical Research Center, Uppsala University, Uppsala, Sweden; 2 Department of Medical Sciences, Cardiology, Uppsala University, Dag Hammarsköldsväg 14B, 75337 Uppsala, Sweden; 3 Department of Internal Medicine, Cardiology, Västerås County Hospital, Västerås, Sweden; 4 Department of Cardiology, Lund University, Lund, Sweden; 5 Department of Clinical Science and Education, Södersjukhuset, Karolinska Institutet, Stockholm, Sweden; 6 Department of Cardiology, Heart Centre, Umeå University, Umeå, Sweden; 7 Faculty of Health and Life Sciences, Linnaeus University, Kalmar, Sweden

**Keywords:** Bare metal stents, Drug-eluting stents, Stent thrombosis, Percutaneous coronary intervention

## Abstract

**Objectives:**

Old-generation drug-eluting coronary stents (o-DES) have despite being safe and effective been associated with an increased propensity of late stent thrombosis (ST). We evaluated ST rates in o-DES, new-generation DES (n-DES) and bare metal stents (BMS) the first year (< 1 year) and beyond 1 year (> 1 year).

**Methods:**

We evaluated all implantations with BMS, o-DES (Cordis Cypher, Boston Scientific Taxus Liberté and Medtronic Endeavor) and n-DES in the Swedish coronary angiography and angioplasty registry (SCAAR) between 1 January 2007 and 8 January 2014 (*n* = 207 291). All cases of ST (*n* = 2 268) until 31 December 2014 were analyzed.

**Results:**

The overall risk of ST was lower in both n-DES and o-DES compared with BMS up to 1 year (n-DES versus BMS: adjusted risk ratio (RR) 0.48 (0.41–0.58) and o-DES versus BMS: 0.56 (0.46–0.67), both *p* < 0.001). From 1 year after stent implantation and onward, the risk for ST was higher in o-DES compared with BMS [adjusted RR, 1.82 (1.47–2.25], *p* < 0.001). N-DES were associated with similar low ST rates as BMS from 1 year and onward [adjusted RR 1.21 (0.94–1.56), *p* = 0.135].

**Conclusion:**

New-generation DES were associated with lower ST rates in comparison to BMS during the first-year post-stenting. After 1 year, n-DES and BMS were associated with similar ST rates.

**Trial Registration:**

This study was a retrospective observational study and as such did not require clinical trial database registration.

**Electronic supplementary material:**

The online version of this article (10.1007/s00392-018-1252-0) contains supplementary material, which is available to authorized users.

## Introduction

Percutaneous coronary intervention (PCI) to treat coronary artery disease has become the most frequently performed therapeutic procedure in medicine [1]. Despite the advantage of stenting, the iatrogenic intimal injury from stenting, not seldom produce a neointimal hyperplasia leading to restenosis and a need for a repeat revascularization [2]. Therefore, drug-eluting stents (DES) coated with antiproliferative agents were developed and demonstrated in randomized trials lower restenosis rates and need for revascularization compared to bare metal stents (BMS) [2–4].

The concern of increased risk of stent thrombosis (ST) with DES, caused by an incomplete neointimal coverage, was raised in the year 2006 after reports from pathoanatomical studies [5], randomized trials [6] and registries [7]. We, therefore, previously evaluated the long-term outcome in all patients who underwent stenting in Sweden between 2003 and 2006 [8, 9]. Compared with bare metal stents, drug-eluting stents were associated with a decrease in the rate of restenosis and but importantly a higher risk of stent thrombosis (ST) and mortality [9]. When the analysis was repeated with an extended patient population, where the course of time had led to a gradual change in patient selection for old-generation drug-eluting coronary stents (o-DES) and adjunct antithrombotic treatment, the association between o-DES and mortality disappeared while the association with ST remained [8].

It is generally accepted that late ST is associated with inadequate stent endothelialization [10]. Whilst the exact cause of poor endothelialization has not been completely elucidated, there are theories that challenge the role of coating polymers due to their potential of evoking localized inflammatory responses [11]. New technologies that followed o-DES such as biodegradable drug-eluting coatings, textured stent surfaces for polymer-free drug–stent attachment and drug-filled reservoirs have aimed at improving long-term outcomes by reducing both late ST and restenosis. Furthermore, neointimal atherosclerotic change, or neoatherosclerosis, is rarely reported and most often occurs beyond 5 years. Nakazawa et al. [12] reported in 2011 that incidence of neoatherosclerosis was significantly higher and occurred earlier in DES than in BMS.

In this study, we evaluated all implantations with BMS, o-DES and n-DES in the nation-wide Swedish coronary angiography and angioplasty registry (SCAAR) between 1 January 2007 and 8 January 2014. We calculated the overall risk of ST in n-DES and o-DES compared with BMS up to 1 year (early and late ST) and more than 1 year after implantation (very late ST).

## Methods

This study was a prospective observational cohort study using data from SCAAR (Swedish Coronary Angiography and Angioplasty Register), a part of the SWEDEHEART registry.

The Swedish nation-wide SCAAR registry captures all coronary angiographies and percutaneous coronary interventions in Sweden. Details about the register were described previously [13]. Since 1 March 2004, the web-based electronic case report form requires that information about restenosis in each and every implanted stent is recorded at the time of any subsequent coronary angiography for any clinical indication.

In our study, we analyzed all implantations (> 1000 implanted individual stents) with BMS, o-DES [Cordis Cypher (Sirolimus, PEVA (polyethylene-co-vinyl acetate) Polymer), Boston Scientific Taxus Liberté (Paclitaxel, SIBS (polyethylene-co-vinyl acetate), Polymer and Medtronic Endeavor (Zotarolimus, Phosphorylcoline Polymer, Medtronic, Inc.)] and n-DES [Medtronic Resolute (Zotarolimus, Bio Linx Polymer, Abbot XienceV (Everolimus, Acrcylic/Fluoro Polymer), Abbot Xience Prime (Everolimus, Acrylic/Fluoro Polymer), Boston Promus (Everolimus, PVDF–HFP (polyvinylidenefluoride–hexafluoropropylene) Polymer), Boston Promus Element (Everolimus, PVDF–HFP Polymer), Biosensors Biomatrix (Biolimus A9, polylactic acid polymer)], between 1 January 2007 and 8 January 2014. All cases of definite ST during this time period were analyzed based on type of assigned stent group (BMS, n-DES or o-DES) and antiproliferative drug. The Ethics Committee at Uppsala University approved the study.

Continuous variables were expressed as means and standard deviations and discrete variables as percentages.

The primary objective was to evaluate occurrence of definite ST in BMS, n-DES and o-DES. The secondary objective was to evaluate the occurrence of definite ST in the different DES according to antiproliferative stent drug. The statistical analysis for ST was performed per stent (not per patient). To compensate for the non-randomized design of this study, multivariate adjustment was performed. The adjusted cumulative risk of ST was calculated using the Cox proportional hazard method.

In the model for calculation of the adjusted relative risk, the following variables that could be potential confounders were included: age, gender, diabetes, hypertension, dyslipidemia, smoking status, clinical indication of the procedure, use of acetyl salicylic acid, GPIIb/IIIa and/or P2Y12 receptor inhibitors at the index procedure, treated vessel, previous myocardial infarction (MI), previous coronary artery bypass grafting (CABG), previous PCI, year of the index procedure, enrolling center, lesion type, bifurcation lesions, restenotic lesions, chronic total occlusions (CTO), stent type, stent diameter, stent length, three-vessel/left main disease, the use of additional stents, and maximal inflation pressure. The statistical interaction between the year of the procedure and the type of stent was assessed in the Cox analysis.

To provide separate descriptions of the relative risks of ST up to 1 year (early and late ST) and after 1 year (very late ST), we performed “landmark analyses” with a prespecified landmark set at 12 months.

All reported *P* values are two sided. All analyses were performed with the use of SPSS statistical software (version 19.0, SPSS Inc., Chicago, IL, USA).

## Results

**Fig. 1:**
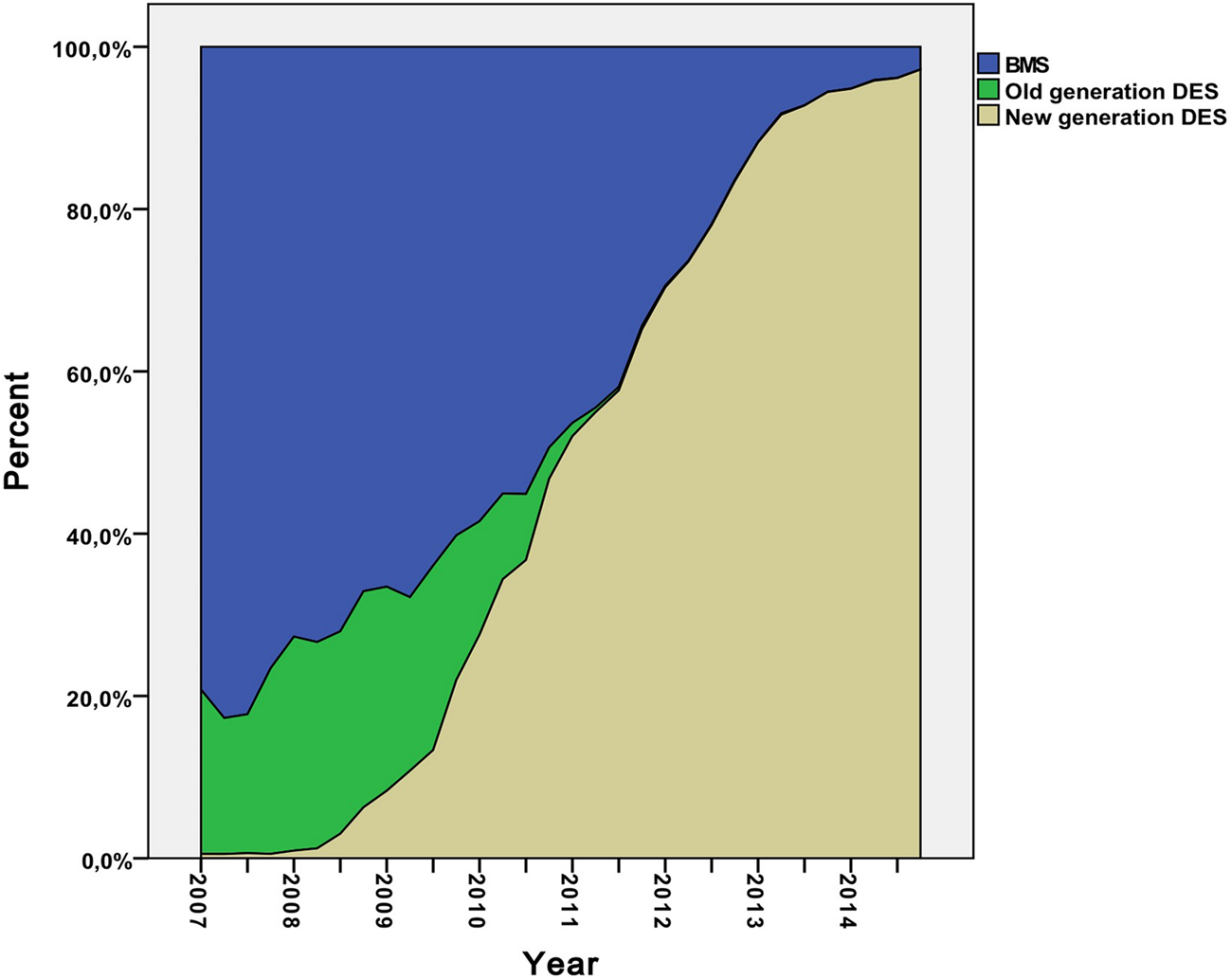
Distribution of the implanted stents: bare metal, new- and old-generation stents during study period

During the study period from year 2007 to 2014, a total of 207,291 stents were implanted, of these were 85,583 BMS, 103,075 n-DES and 18,633 o-DES. The distribution of stent types during the observation period is shown in Fig. 1.

The background and procedural characteristics of the patients treated with the stents analyzed are shown in Table 1. Bare metal stents as compared to DES were more likely to have been used in STEMI patients and less likely in patients with previous PCI and diabetes. Within the DES groups (n-DES versus o-DES), there were no major differences in patient and procedural characteristics, except a shift from clopidogrel to ticagrelor before PCI and a less frequent use of GPIIb/IIIa-inhibitors in n-DES compared to o-DES (Table 1).

**Table 1:** Baseline and procedural characteristics for stent groups treated with bare metal stents, new- and old-generation stents

Variable (n %)	BMS (*n* = 85,583)	n-DES (*n* = 103,075)	o-DES (*n* = 18,633)
Women	23,355 (27.3)	25,387 (24.6)	4901 (26.3)
Age, yrs	67.9 ± 11	67.2 ± 10.7	66.0 ± 10.4
*Indication for the procedure*			
Stable angina	15,705 (18.4)	29,605 (28.7)	6534 (35.1)
ST-elevation MI	28,395 (33.2)	19,134 (18.6)	2084 (11.2)
Non-ST-elevation MI/unstable angina	39,024 (45.6)	51,375 (49.8)	9640 (51.7)
*Coexisting conditions*			
Hypertension	46,113 (53.9)	66,999 (65.0)	11,571 (62.1)
Diabetes mellitus	13,901 (16.2)	24,042 (23.3)	5215 (28.0)
Hypercholesterolemia	39,759 (46.5)	59,814 (58.0)	12,295 (66.0)
Smoker status: current	18,140 (21.2)	18,326 (17.8)	2744 (14.7)
Smoker status: former > 1 month	29,360 (34.4)	40,321 (39.1)	7250 (38.9)
Previous MI	20,209 (23.6)	31,744 (30.8)	7195 (38.6)
Previous PCI	16,051 (18.8)	32,313 (31.3)	7844 (42.1)
Previous CABG	7253 (8.5)	11,095 (10.8)	2832 (15.2)
*Procedural characteristics*			
No. of stents per procedure	1.88 ± 1.07	2.25 ± 1.3	2.24 ± 1.25
Stent diameter (mm)	3.1 ± 0.53	2,97 ± 0.52	2.7 ± 0.50
Total stent length (mm)	17.0 ± 5.6	20.1 ± 7.7	19.2 ± 7.1
Treated vessel: RCA	30,617 (35.8)	29,061 (28.2)	4788 (25.7)
Treated vessel: left main	1158 (1.4)	3544 (3.4)	653 (3.5)
Treated vessel: LAD	32,945 (38.5)	45,238 (43.9)	8260 (44.3)
Treated vessel: LCX	18,370 (21.5)	22,381 (21.7)	4061 (21.8)
Treated vessel: CABG graft	2493 (2.9)	2851 (2.8)	871 (4.7)
Bifurcation lesion	6611 (7.7)	13,532 (13.1)	2556 (13.7)
Three-vessel disease	16,965 (19.8)	21,224 (20.6)	4060 (21.8)
*Medications before PCI*			
ASA	78,387 (91.6)	97,661 (94.7)	17,911 (96.1)
Clopidogrel	67,707 (79.1)	53,609 (52.0)	16,099 (86.4)
Ticagrelor	4631 (5.4)	41,347 (40.1)	32 (0.2)
Prasugrel	3010 (3.5)	4707 (4.6)	81 (0.4)
*Medications under PCI*			
GP IIb/IIIa	17,529 (20.5)	6235 (6.0)	2248 (12.1)
Heparin	50,617 (59.1)	71,090 (69.0)	13,186 (70.8)
LMWH	7949 (9.3)	5554 (5.4)	1621 (8.7)
Bivalirudin	25,904 (30.3)	25,574 (24.8)	3625 (19.5)

Values are *N* (%) or mean ± SD

*BMS* bare metal stent, *n-DES* new-generation drug-eluting stent, *o-DES* old-generation drug-eluting stent, *MI* myocardial infarction, *CABG* coronary artery bypass grafting, *PCI* percutaneous coronary stenting, *ASA* acetylsalicylic acid, *GP* glycoprotein, *LAD* left anterior descending artery, *LCX* left circumflex artery, *LMWH* low molecular weight heparin, *PCI* percutaneous coronary intervention, *RCA* right coronary artery

**Fig. 2:**
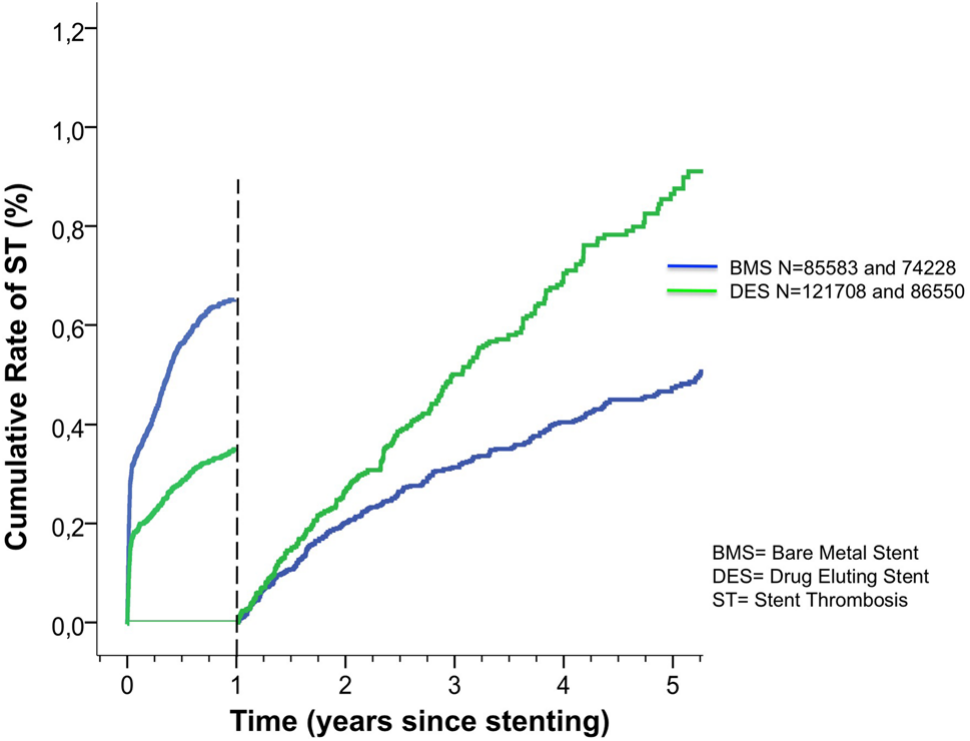
Cumulative rate of stent thrombosis (unadjusted) in bare metal and drug-eluting stents implanted

A total of 2268 cases of ST occurred during the study period. The risk of stent thrombosis was lower in DES (both n-DES and o-DES) compared to BMS up to 1 year {DES versus BMS: adjusted risk ratio (RR) [0.51 (0.45–0.59) *p* < 0.001]} but for ST {from 1 year and onward the risk of ST was higher with DES than with BMS [DES versus BMS: (1.55 (1.28–1.87) *p* < 0.001)]} (Fig. 2).

**Fig. 3:**
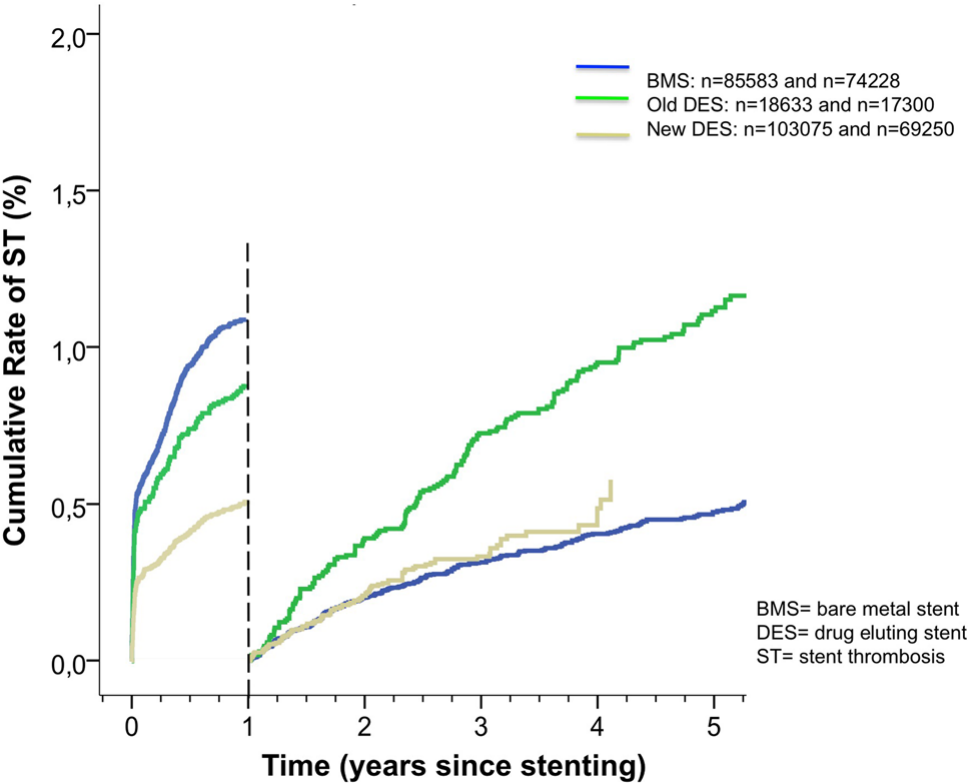
Cumulative rate of stent thrombosis (unadjusted) in bare metal, new- and old-generation stents

The overall risk of ST was lower in both n-DES (*n* = 103 075) and o-DES (*n* = 18 633) compared with BMS (*n* = 85 583) up to 1 year [n-DES versus BMS: adjusted risk ratio (RR) 0.48 (0.41–0.58) and o-DES versus BMS: 0.56 (0.46–0.67), both *p* < 0.001] (Fig. 3; Table 2).

**Table 2:** Adjusted risk ratios for stent thrombosis in bare metal stents, new-generation and old-generation drug-eluting stents

	Definite ST up to 1 year	Definite ST from 1 year and onward
o-DES versus BMS	0.56 (0.46–0.67)*	1.82 (1.47–2.25)*
n-DES versus BMS	0.48 (0.41–0.58)*	1.21 (0.94–1.56)
n-DES versus o-DES	0.87 (0.69–1.09)	0.67 (0.51–0.87)*

Risk ratios and 95% confidence intervals for definite stent thrombosis

*o-DES* old-generation drug-eluting stents, *n-DES* new-generation drug-eluting stents, *BMS* bare metal stents, *ST* stent thrombosis

*Significant comparisons (*p* < 0.05)

From 1 year after stent implantation and onward, the risk for ST was higher in o-DES (*n* = 17 300) compared with BMS (*n* = 74 228) [adjusted RR, 1.82 (1.47–2.25), *p* < 0.001]. New-generation DES (*n* = 69 250) were associated with similar low ST rates as BMS from 1 year and onward [adjusted RR 1.21 (0.94–1.56), *p* = 0.135].

Compared to o-DES, n-DES showed a lower risk for stent thrombosis both up to 1 year [adjusted RR 0.87 (0.69–1.09), *p* = 0.232] and from 1 year onwards [adjusted RR 0.67 (0.51–0.87), *p* = 0.003] (Fig. 3; Table 2).

We performed a sensitivity analysis omitting the Endeavor zotarolimus-eluting stent from the n-DES group with similar results to the main analysis (Supplemental Table 1).

**Fig. 4:**
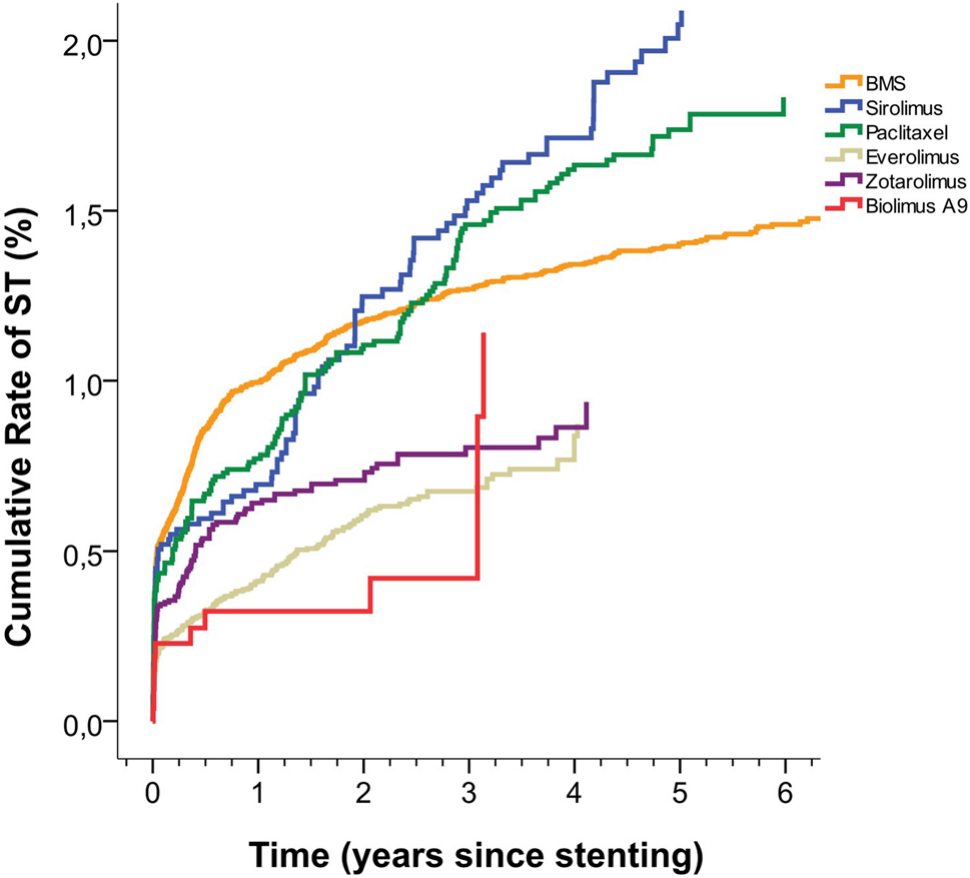
Cumulative rate of stent thrombosis (adjusted) in bare metal stents and drug-eluting stents according to different stent drugs

When we analyzed ST rates in stents according to drug coating, both sirolimus- and paclitaxel-coated stents were associated with higher stent thrombosis rates from 1 year and onward compared to BMS [adjusted RR 2.00 (1.41–2.83) and 1.54 (1.14–2.08) for sirolimus and paclitaxel, respectively]. During the first year, both sirolimus- and paclitaxel-coated stents were associated with lower ST rates than BMS. Everolimus-, zotarolimus- and biolimus-coated stents were associated with lower ST rates both up to 1 year and from 1 year and onward (Fig. 4; Table 3).

**Table 3:** Adjusted risk ratios for stent thrombosis in bare metal stents versus drug-eluting stents according to drug-eluting stent drug

	Definite ST up to 1 year	Definite ST from 1 year and onward
Sirolimus versus BMS	0.71 (0.53–0.95)*	2.00 (1.41–2.83)*
Paclitaxel versus BMS	0.78 (0.61–0.98)*	1.54 (1.14–2.08)*
Everolimus versus BMS	0.41 (0.35–0.48)*	1.05 (0.76–1.46)
Zotarolimus versus BMS	0.64 (0.52–0.77)*	0.84 (0.55–1.29)
Biolimus versus BMS	0.33 (0.17–0.67)*	1.12 (0.39–3.24)

Risk ratios and 95% confidence intervals for definite stent thrombosis

*BMS* bare metal stents, *ST* stent thrombosis

*Significant comparisons (*p* < 0.05)

## Discussion

In this study, we report from a large cohort of unselected consecutive patients treated with coronary stents at all interventional centers in Sweden that n-DES are associated with reduced ST rates during the first year after implantation. Importantly, in contrast to o-DES, n-DES were not associated with higher very late ST rates after 1 year compared to BMS. This benefit compared to BMS and o-DES, seemed to be maintained during the follow-up period of up to 5 years. This non-randomized comparison between the stent types was adjusted for all available confounders but there is always a possibility of selection bias because of unknown confounders. Nonetheless, the reliability of our results is strengthened by the fact that all ST cases have been angiographically assessed, registry source-data verification (showing a 95% correspondence with patients’ hospital records) and the use of definite, angiographically proven ST and not Academic Research Consortium (ARC) probable/possible ST as the only endpoint measure.

The technical development of DES has aimed at reducing the trade-off between delayed coronary vessel healing and restenosis, both of which contribute to stent thrombosis risk. New-generation DES as compared to o-DES have thinner polymer, reduced strut thickness and alternative drugs that may modulate the long-term risk of ST [14–18].

Most of the n-DES received CE (conformité européenne) mark approval based on results from non-inferiority trials compared with o-DES [19, 20]. Although not individually powered to show differences in ST, recent data from direct comparisons between zotarolimus-eluting and everolimus-eluting stents have demonstrated similar safety and efficacy throughout 4 years [21, 22]. Furthermore, a pooled analysis of two trials evaluating BMS versus cobalt–chromium everolimus-eluting or biolimus A9-eluting stents showed a reduced risk of definite ST up to 1 year compared to BMS [23–25]. In a randomized, single-blind, non-inferiority study comparing a bioabsorbable polymer everolimus-eluting stent with an everolimus-coated stent, rates of ST and target lesion failure at 1 year were similar [15]. In a large network metaanalysis by Palmerini et al. [26], including fifty-one trials and 52,158 patients with a median follow-up of 3.8 years, n-DES showed improved safety as compared to o-DES.

The risk of ST is strongly determined by antiplatelet drug response, duration of antiplatelet therapy, burden of cardiovascular risk and technical factors that influence the success of coronary stent implantation [27, 28]. In acute coronary syndrome (ACS) patients, both prasugrel and ticagrelor have demonstrated greater efficacy in preventing ST compared to clopidogrel [29, 30]. During the time period of the present study, a shift from clopidogrel to ticagrelor as the most widely used P2Y12-receptor antagonist in the treatment of ACS in Sweden occurred [31]. In this study, also beyond the standard and in Sweden widely used dual antiplatelet treatment duration post-ACS of 1 year, we showed that n-DES were associated with lower rates of ST (> 1 year) compared to BMS. Also in patients with more complex coronary artery disease (SYNTAX score > 11 vs. ≤ 11) recent data from four all-comer trials showed improved clinical outcomes, including reduced ST rates, with n-DES as compared to o-DES [32]. In the recently published Norwegian Coronary Stent Trial (NORSTENT), lower rates of repeat revascularization but no difference in death or nonfatal spontaneous MI rates were shown in patients receiving DES as compared to BMS [33]. Interestingly, overall rates of definite ST at 6-year follow-up was similar to those in our study and also appeared to be lower in DES compared to BMS patients [0.8% for DES and 1.2% for BMS (*p* = 0.0498)]. The majority of DES implanted were everolimus (82.9%) followed by zotarolimus (13.1%) eluting and no o-DES were implanted. The results support our finding of a long-term durability of improved outcomes with new-generation drug-eluting stents.

## Limitations

Despite the use of statistical adjustments, differences in baseline characteristics or selection criteria that might not have been recorded could remain and influence the results. Also, changes in event rates over time could have been influenced by the smaller number of patients with drug-eluting stents early in the study period. The analysis of ST according to antiproliferative agent is limited by the fact that differences between stents are based on all three components of the stent (i.e., backbone, coating and antiproliferative agent). The database did not contain information on the duration and type of the antiplatelet treatment. Therefore, data for the first year are less reliable also due to the fact that the indication for the index procedure (stable versus acute indication) and antithrombotic treatment could influence ST rates.

## Conclusions

In this large study of 207,291 stents with complete long-term follow-up, new-generation DES were associated with ST rates compared to BMS up to 1 year and after 1 year (very late ST) compared to old-generation DES. Our results are consistent with the previous report on n-DES from SCAAR [34, 35] and confirm a higher performance of n-DES in an even larger population and a longer follow-up.

## Electronic supplementary material

Below is the link to the electronic supplementary material.

Supplementary material 1 (DOCX 12 KB)
