# Supplementary material for: Stent thrombosis rates the first year and beyond with new- and old-generation drug-eluting stents compared to bare metal stents
Source: Clin Res Cardiol. 2018 Apr 17;107(9):816–23. doi: 10.1007/s00392-018-1252-0 (PMC6105307; doi:10.1007/s00392-018-1252-0)
Supplement: Supplementary file 1 — Supplementary material 1 (DOCX 12 KB) [file 392_2018_1252_MOESM1_ESM.docx]

**Stent thrombosis rates the first year and beyond with new and old generation drug eluting stents compared to bare metal stents**

*Varenhorst: Drug eluting stents and stent thrombosis*

**Supplemental Table 1**

Adjusted risk ratios for stent thrombosis in bare metal stents, new generation- and old generation drug eluting stents (the zotarolimus-eluting stent Endeavor removed from the analysis).

|  | Definite ST up to one year | Definite ST from one year and onward |
| --- | --- | --- |
| o-DES vs. BMS | 0.50 (0.40-0.62) | 2.15 (1.73-2.68) |
| n-DES vs. BMS | 0.48 (0.41-0.58) | 1.23 (0.96-1.58) |
| n-DES vs. o-DES | 0.97 (0.76-1.24) | 0.57 (0.44-0.75) |

o-DES: old generation drug eluting stents, n-DES: new generation drug eluting stents, BMS: bare metal stents, ST: stent thrombosis. Risk ratios and 95% confidence intervals for definite stent thrombosis.

o-DES up to one year n=15253 and from one year and onwards n=14245
